# The Johns Hopkins Hospital Reports

**Published:** 1898-01

**Authors:** 


The Johns Hopkins Hospital Reports. Volume VI. Baltimore: The Johns Hopkins Press. 1897.

This excellent report, containing 414 pages, is devoted to several different departments of the hospital and shows what careful, painstaking work is done by the heads of the various departments. The volume contains a masterful study of the lesions produced by the action of certain poisons on the cortical nerve cell by Henry J. Berkley, illustrated with some of the most beautiful photomicrographs of nerve cells that the reviewer has ever seen.

The report in pathology embraces several sub-reports by Drs. Thomas S. Cullen, G. P. Wilkins, William D. Barker and Simon Flexner, as Fatal puerperal sepsis due to introduction of an elm tent; Pregnancy in a rudimentary uterine horn, rupture, death; Probable migration of ovum and spermatozoa; Adeno-myoma uteri diffusum benignum; A bacteriological and anatomical study of the summer diarrhea of infants ; and The pathology of toxalbumin intoxication.

The illustrations, twenty-four plates, many colored, are models of their kind and add materially to the value of the reports.

